# The role of provincial health administration in supporting district health management teams in the Democratic Republic of Congo: eliciting an initial programme theory of a realist evaluation

**DOI:** 10.1186/s12961-024-01115-9

**Published:** 2024-02-20

**Authors:** Samuel Bosongo, Zakaria Belrhiti, Faustin Chenge, Bart Criel, Yves Coppieters, Bruno Marchal

**Affiliations:** 1 Faculté de Médecine et Pharmacie, Université de Kisangani, Kisangani, Democratic Republic of Congo; 2 École de Santé Publique, Université Libre de Bruxelles, Brussels, Belgium; 3 Institute of Tropical Medicine, Antwerp, Belgium; 4 Centre de Connaissances en Santé en République Démocratique du Congo, Kinshasa, Democratic Republic of Congo; 5 Mohammed VI University of Sciences and Health (UM6SS), Casablanca, Morocco; 6 Centre Mohammed VI de la Recherche et Innovation (CM6), Rabat, Morocco; 7 Ecole de Santé Publique, Faculté de Médecine, Université de Lubumbashi, Lubumbashi, Democratic Republic of Congo

**Keywords:** Technical support, District health management teams, Provincial health administration, Democratic Republic of Congo, Realist evaluation

## Abstract

**Background:**

In 2006, the Ministry of Health in the Democratic Republic of Congo designed a strategy to strengthen the health system by developing health districts. This strategy included a reform of the provincial health administration to provide effective technical support to district health management teams in terms of leadership and management. The provincial health teams were set up in 2014, but few studies have been done on how, for whom, and under what circumstances their support to the districts works. We report on the development of an initial programme theory that is the first step of a realist evaluation seeking to address this knowledge gap.

**Methods:**

To inform the initial programme theory, we collected data through a scoping review of primary studies on leadership or management capacity building of district health managers in sub-Saharan Africa, a review of policy documents and interviews with the programme designers. We then conducted a two-step data analysis: first, identification of intervention features, context, actors, mechanisms and outcomes through thematic content analysis, and second, formulation of intervention–context–actor–mechanism–outcome (ICAMO) configurations using a retroductive approach.

**Results:**

We identified six ICAMO configurations explaining how effective technical support (i.e. personalised, problem-solving centred and reflection-stimulating) may improve the competencies of the members of district health management teams by activating a series of mechanisms (including positive perceived relevance of the support, positive perceived credibility of provincial health administration staff, trust in provincial health administration staff, psychological safety, reflexivity, self-efficacy and perceived autonomy) under specific contextual conditions (including enabling learning environment, integration of vertical programmes, competent public health administration staff, optimal decision space, supportive work conditions, availability of resources and absence of negative political influences).

**Conclusions:**

We identified initial ICAMO configurations that explain how provincial health administration technical support for district health management teams is expected to work, for whom and under what conditions. These ICAMO configurations will be tested in subsequent empirical studies.

**Supplementary Information:**

The online version contains supplementary material available at 10.1186/s12961-024-01115-9.

## Introduction

In an ever-evolving world, health systems are under pressure. They have to perform better to meet the population’s expectations while dealing with various challenges, such as the ageing population, climate change, the double burden of infectious and non-communicable diseases, re-emerging diseases and violent conflicts [1]. However, health systems in most sub-Saharan countries remain weak and fragile, and they struggle to progress towards universal health coverage [2]. While the WHO acknowledges that health system strengthening is the principal means to achieve universal health coverage [3], little is known about how best to do so [2]. Decentralisation is a widespread health sector reform in sub-Saharan Africa that aims to improve health systems’ performance in terms of access, quality, equity, efficiency and financial protection [2, 4, 5]. However, better information and evidence are still needed to guide this reform [2].

The health system in the Democratic Republic of Congo (DRC)—whose overall structure is outlined in Box 1—has been ranked among the worst-performing in Africa for almost three decades [6–8]. This situation is partly due to persistent socio-political crises and systematic underfunding of the health sector [9]. In response to these crises, a range of emergency interventions have been implemented by humanitarian agencies, and much space was given to vertical disease control programmes. However, instead of strengthening a comprehensive primary health care system, the foundation of the DRC’s national health policy, these programmes were selective and often a source of disruption and distortion of the already weakened regular health system [9–11]. Furthermore, a massive expansion of the number of universities led to an uncontrolled increase in medical doctors [12] and a booming but poorly regulated private-for-profit sector [9, 10]. This situation contributed to increasing the disintegration, fragmentation, lack of coordination and inefficiency of the health system [9].

Box 1. The overall governance structure of the health system in the DRC The health system in the DRC is structured into three levels: national, provincial and operational. At the national level, the National Ministry of Health oversees the General Secretariat for Health and the General Health Inspectorate, each with its central directorates. They are responsible for setting norms, policies, and guidelines and monitoring their implementation at the sub-national levels.

The provincial level includes the Provincial Ministry of Health, the Provincial Health Division (referred to in this paper as Provincial Health Administration) and the Provincial Health Inspectorate, each with its own offices. The provincial health division provides technical support to health districts, while the provincial health inspectorate ensures the enforcement of national-level norms, policies and guidelines.

The operational level consists of health districts where national health policies are implemented. It comprises two specific yet complementary healthcare levels, overseen by the district health management team. The first level includes a network of first-line health facilities that provide primary care to the population. The second level comprises one or more hospitals that offer more technical or specialised care. The district health management team includes healthcare professionals with management or administrative positions. They have diverse professional backgrounds, including physicians, nurses, pharmacists, nutritionists and administrators, and perform different roles, such as district medical officers, hospital directors, clinicians, nursing officers and nurse supervisors.

These three levels are linked hierarchically so that the lower levels are accountable to the direct higher level. Although the provincial and operational levels are supposed to operate in a decentralised fashion, they need more decision space, especially in human resource management and financial resource mobilisation and allocation.

In response to this situation, the Ministry of Health (MoH) developed the Health System Strengthening Strategy in 2006 [11] and updated it in 2010 [10]. This strategy followed the adoption of the new constitution, which embodies the principle of decentralisation [13]. A key pillar of this strategy is strengthening the health districts, considered the essential lever for strengthening the health system [10, 11]. It recommended restructuring the Provincial Health Administration (PHA) so that it can provide effective technical support to district health management teams (DHMTs) to develop their leadership and management capacities. The PHA reform involved a functional, a structural and a cultural reorganisation. The functional reorganisation separated the inspection and control function from technical support to the health districts. The structural reorganisation involved moving from 13 offices and multiple vertical programmes to four core functions corresponding to four PHA offices: (1) technical support for health districts office, (2) health information, communication and research office, (3) inspection and control office and (4) resource management office. Two offices were added: the public hygiene office and the health sciences education office. In addition to these administrative offices, the PHA office also includes a number of thematic working groups. They are ad hoc functional bodies (or taskforces) where PHA staff from all offices can meet to discuss and reflect on specific issues, such as technical support to health districts, health information management, medicine supplies to health districts, health financing coordination and epidemiological surveillance. They are designed to promote synergy among staff, encourage participation in decision-making and foster learning. The cultural shift aimed to gradually transition from a hierarchical to an adhocracy culture [14].

Technical support to health districts is the central role of the PHA office. It is supposed to enhance the leadership and management capacities of DHMT members to improve the health district’s performance and, ultimately, the overall health outcomes of the population. These capacities include coordinating stakeholders, planning and budgeting, monitoring and evaluation, training and supervising health workers, managing health system information, conducting epidemiological surveillance, managing resources (human, financial, material and medicines) and conducting operational and action research [15]. Technical support is to be provided through facilitative supervision, coaching and problem-solving support. The PHA reform assigned the technical support role to experienced PHA staff with public health and district management backgrounds. Their number depends on the number of health districts in the province and the availability of qualified staff at the PHA offices. In practice, each PHA staff member is responsible for supporting two to four health districts. They provide technical support to DHMT members through field visits, which are scheduled based on issues identified by PHA staff through analysis of data, plans and activity reports of each health district or based on concerns raised by the DHMT members.

Between 2008 and 2011, pilot action research conducted in the provinces of North Kivu and Eastern Kasai to test PHA reorganisation pointed to positive results in terms of an adequate structure of PHA for providing better support to health districts [14]. Consequently, the PHA reform was rolled out in all 26 provinces of the DRC between 2014 and 2015. Since the rollout of the PHA reform, two studies on the technical support of DHMT members have reported contrasting results. One study found that DHMT members greatly appreciated technical support in one province [16]. However, in another province, there was a reported lack of a clear conceptual model to guide the operationalisation of this support [17]. This divergence may be partially explained by the research methods used (quantitative versus mixed methods) and the study contexts (rural and urban versus urban only). However, it also points to how technical support is being implemented across different provinces and how actors’ perceptions and responses influence this implementation process.

As a capacity building intervention, the technical support from the reformed PHAs to DHMTs is a complex intervention in districts which can be considered as complex systems [18]. In such systems characterised by continuous interactions between actors, their organisation and their environment, the outcomes (improved leadership and management capacities of DHMT members) of the technical support from the PHAs (intervention) cannot fully be predicted. Moreover, more knowledge about how such capacity building interventions improve the performance of health workers in low- and middle-income countries, such as the DRC, is needed [18]. In this paper, we present how we have elicited the initial programme theory of technical support, which is the first step of the realist evaluation we are conducting (explained below).

## Methodology

### The methodological approach

Our overall methodological approach is realist evaluation (RE). RE is a theory-driven evaluation approach that seeks to understand why and how a programme works, for whom, and in what circumstances [19, 20]. By answering these questions, RE attempts to provide a plausible causal account of how the interaction of actors with an intervention triggers mechanisms that lead to outcomes within a given context. This context-sensitive approach is well suited for evaluating complex interventions, such as capacity building within complex systems (for example health districts) [18].

The realist approach considers social programmes to be theories, active and embedded in social systems [20]. This view of social programmes has methodological implications for RE. First, as programmes are theories incarnate, realist researchers are tasked with eliciting, testing and refining the underlying programme theories [20]. A programme theory is a set of assumptions explaining how an intervention brings about changes (intended or not) by activating mechanisms among actors in a given context. Second, programmes work through people’s reasoning. In other words, programmes do not bring about changes but the actors do through their reasoning and responses to the resources provided by a programme. People’s reasoning and resources are called mechanisms and generate outcomes in a particular context [21–23]. Hence, realist evaluators seek to identify these underlying generative mechanisms. Finally, programmes are open systems embedded in social systems that continuously interact and influence each other. Thus, an essential requirement of RE is to take heed of the social environment (or context) surrounding programmes as it conditions the firing of mechanisms [20, 24–26].

In practice, RE begins and ends with a theory [27, 28]. It has three main stages: (1) eliciting the initial programme theory (IPT), (2) testing the IPT through empirical studies and (3) refining the IPT based on the results of empirical studies. The resulting refined programme theory is to be further tested and refined in new studies. Realist researchers use the context–mechanism–outcome (CMO) configuration as a heuristic tool. In this study, we use a fine-tuned variant, the intervention–context–actors–mechanisms–outcomes (ICAMO) configuration, to better differentiate intervention from context and emphasise the role of different actors in the change processes [29]. In realist evaluation literature, intervention and context are sometimes conflated to the extent that aspects of ‘intervention’ are often reported as ‘contextual factors’. However, context and intervention are separate conceptual and analytical entities in realist evaluation. Therefore, it is important to provide a detailed description of contextual factors and intervention components or features. This will help to identify which contextual factors influence which intervention component, thus triggering the mechanisms that lead to the outcomes. Furthermore, when implementing an intervention, it is essential to recognize that various stakeholders have unique roles, perspectives and reasoning. Emphasizing these differences can provide valuable insights into the ‘for whom’ question in realist evaluation, and the ICAMO configuration is a heuristic that draws the attention of the analysts to these issues [30].

### Data collection

**Fig. 1:**
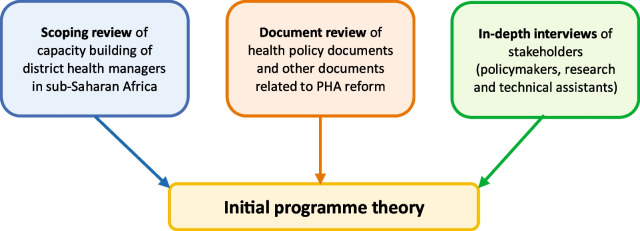
The process of IPT development

We collected data through a scoping review of the capacity building of district health managers in sub-Saharan Africa [31], a review of documents related to the PHA reform in the DRC and in-depth interviews with stakeholders of the PHA reform (Fig. 1).

#### Scoping review

We conducted a scoping review to describe how capacity building programmes for district health managers are designed, delivered and evaluated in sub-Saharan Africa. We focused on identifying the underlying assumptions or theories behind these programmes. We searched for relevant studies through five electronic databases (PubMed, Health Systems Evidence, Wiley Online Library, Cochrane Library and Google Scholar), grey literature and citation tracking. We included all primary studies reporting leadership or management capacity building of district health managers in sub-Saharan Africa, written in English or French, and published between 1 January 1987 and 13 October 2022. Further details on the scoping review can be found elsewhere [31].

#### Document review

We aim to understand the process of PHA reform in the DRC and to collect general information about the implicit logic model of the technical support from PHA staff to DHMT members. The documents were from different sources, including the MoH, supporting partner organisations and previous studies in the DRC. We included 21 documents based on their relevance, i.e. documents that provide appropriate information related to the PHA reform and technical support to health districts [32, 33]. The type and description of the included documents are summarised in the Additional file 1.

#### In-depth interviews

To gain a better understanding of how and why the intervention would bring about expected changes, we conducted interviews with stakeholders involved in the design of the PHA reform (Table 1). We purposively identified 15 potential respondents and contacted them via email to invite them for an interview. An information sheet on the study was provided. Thirteen participants accepted, and ten respondents were interviewed. Three accepted but could not be interviewed due to persistently conflicting agendas. We used a piloted interview guide with open-ended questions (Additional file 2). Questions were related to the general process of PHA reform in the DRC, the expected outcomes from the reform and the technical support, the process of technical support and the contextual factors that may influence the technical support process and outcomes. All interviews were conducted in French by the first author (a male medical doctor from the DRC and a PhD student trained in qualitative methods) and online using Teams, Zoom and WhatsApp applications in March 2023. Nine interviews were audio recorded and lasted 50 min on average, and one interview was conducted through WhatsApp chat due to poor internet connection. The interviews were transcribed verbatim and sent to the participants for comment and/or correction. In addition to audio-recorded interviews, we sent follow-up questions via email to four respondents to gain a deeper understanding of certain issues that arose during our data analysis. Saturation was achieved after the ten interviews and follow-up questions. Therefore, no further interviews were conducted with other potential participants identified to replace those who were not available due to persistent conflicting agendas.

**Table 1:** Characteristics of respondents

Participants	Initial interviews (*n* = 10)	Follow-up interviews (*n* = 4)
Role		
MoH staff	4	2
Technical and financial partners	4	1
Researchers	2	1
Qualification		
Physicians	9	4
Psychologist	1	0
Age		
< 50 years	0	0
≥ 50 years	10	4
Gender		
Male	9	4
Female	1	0

### Data analysis

**Fig. 2:**
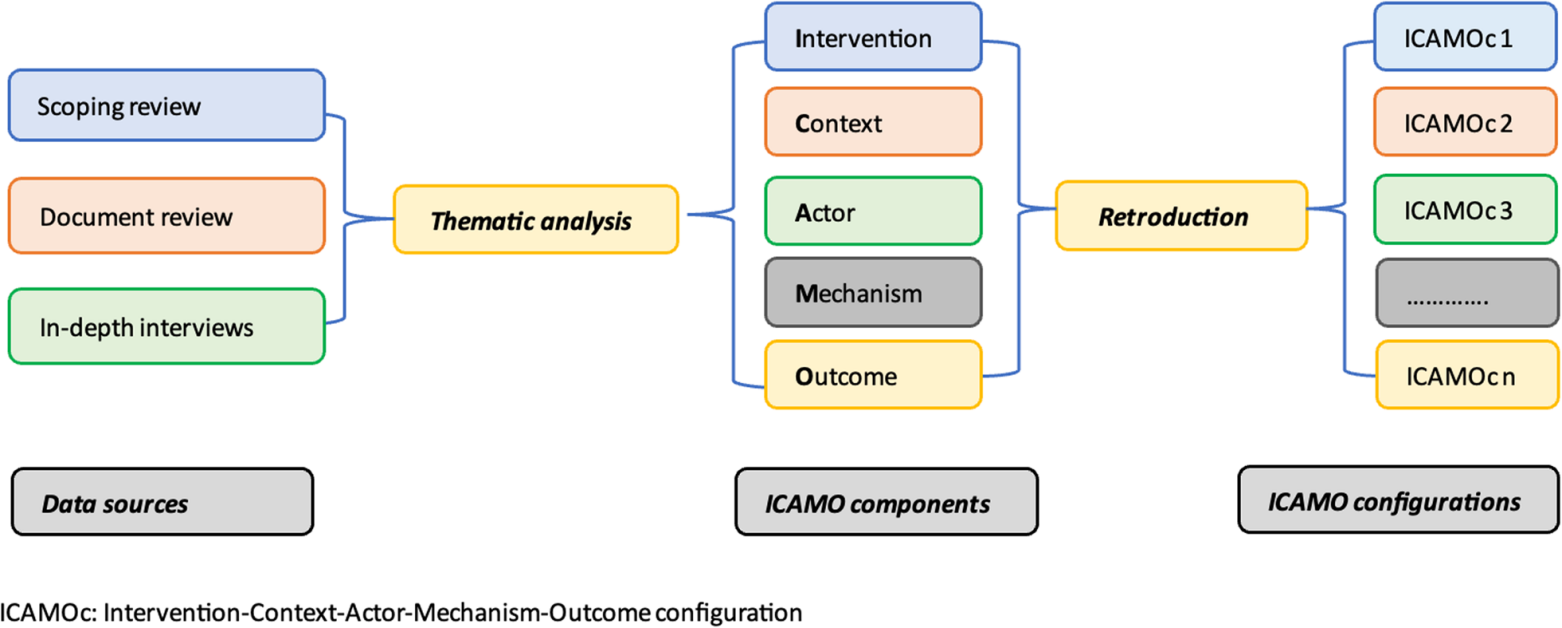
Data analysis process

The analysis of the data from the three sources was combined. We analysed the data in two steps: (1) identification of ICAMO components and (2) formulation of ICAMO configurations (Fig. 2).

#### Step 1. Identification of ICAMO components

To manage the data, we imported the interview transcripts, documents, and results of the scoping review into N-Vivo 14. After familiarisation with the transcripts and the documents through multiple readings, the first author performed a framework analysis [34], applying thematic content analysis to identify themes related to the intervention, context, actors, mechanisms and outcomes. We performed both manifest and latent content analysis [35]. We started the coding with a focus on the manifest content, focusing on the terms and concepts used by the respondents to describe elements of intervention, context, actors, mechanisms and outcomes. In a second reading of the interviews, we focused on the latent content, probing for the interpretations of the respondents, and more specifically on whether and how they identified causal explanations. An a priori codebook developed by the research team was used (Table 2) [32]. The following questions guided this step:

- What are the expected outcomes of the technical support of DHMT members by PHA staff?
- What are the components or features of this technical support? How should these components be carried out?
- Who are the actors involved in implementing technical support?
- What contextual factors can facilitate or hinder actors in taking up technical support?
- What possible mechanisms can be triggered by technical support for producing the reported outcomes?

**Table 2:** Codebook

ICAMO elements		Definitions	Coding rules
Intervention		A combination of policy or programme components or strategies, especially those meant to change people’s behaviour [36]	Use this code to document any intervention features, component or strategy planned or used to achieve the expected outcomes
Context		Any pre-existing social, economic, cultural, political or other environmental factor that may influence the implementation and/or the actors and that may shape the outcomes	Use this code to identify social, economic, cultural, political or other environmental factors that may enable or hinder the expected outcomes
Actors		The people, groups and institutions who are addressed by the intervention and who are central to its adoption and implementation	Use this code to capture any attributes (background, experience, knowledge, skills and attitude), actions or actual practice of an individual, group or institution
Mechanisms		People’s reasoning and reactions to resources made available by the intervention, triggered in specific contexts [22]	Use this code to capture why actors behave or act to achieve or not the expected outcomes
Outcomes	Short-term or immediate	The immediate effect of programme activities in terms of knowledge, skills or awareness [36]	Use this code to document changes in the intervention direct beneficiaries’ knowledge, skills or awareness
	Medium-term or intermediate	Behavioural changes that follow the immediate knowledge and awareness changes [36]	Use this code to capture the changes that follow the changes in knowledge, skills or awareness, such as changes in district performance
	Long-term	Distal changes, such as health status and impact on communities or the health system [36]	Use this code to document the impact of the intervention

#### Step 2. Formulation of ICAMO configurations

In the second step, we used a retroductive approach to identify the links between the intervention, context, actors, mechanisms and outcomes, i.e. the ICAMO configurations. The ICAMO configuration is a plausible causal pathway that explains how actors deal with intervention components within a given context, which activates mechanisms that lead to outcomes. Retroduction is a mode of inference that seeks to ‘unearth the activated mechanisms’ [25, 26]. In practice, we started from the expected outcomes and worked backwards through data to determine the intervention component, plausible mechanisms and contextual conditions that could cause them. The guiding questions at this step were as follows:

- What outcome(s) can be linked to the implementation of the component(s) of technical support of DHMT members by PHA staff?
- What mechanism(s) can link the outcome(s) to the component(s) of technical support of DHMT members by PHA staff?
- What contextual condition(s) can facilitate or hinder the activation of such mechanism(s)?
- Are there alternative explanations?

### Ethical considerations

The research protocol was approved by the Institutional Review Board (IRB) of the Institute of Tropical Medicine, Antwerp (reference IRB no. 1654/22) and the Medical Ethics Committee of the University of Lubumbashi (reference no. UNILU/CEM/005/2023). Prior to the interviews, we sent the study information sheet and the informed consent form to the potential participants and obtained their agreement by e-mail. The information sheet contained information about the study’s objectives, the voluntary nature of the participation, confidentiality measures and benefits and risks associated with the study. To ensure confidentiality, we pseudonymised the data and gave each participant a code. All data were stored in a password-protected drive.

## Results

In this section, we first present the individual components of ICAMO, followed by the ICAMO configurations.

### ICAMO components

In this subsection, we begin with the expected outcomes, the starting point of our retroductive approach described above.

#### Outcomes

The expected immediate outcome is a competent DHMT. Technical support to health districts is meant to strengthen the competencies of DHMT members in performing their managerial tasks (coordination, planning, monitoring, evaluation, supervision, managing resources, etc.) and clinical functions, as explained in the excerpts below:

“The PHA staff should strengthen the DHMT members’ managerial functions in order to enable them to develop a functional local health system”. [DR_12_].

“The clinical function is an essential aspect that is often overlooked, yet this is the raison d'être of any healthcare system. A DHMT must have clinical competencies to supervise health facilities effectively”. [IDI_1_]

The intermediate outcome is to improve the performance of health districts. A competent DHMT is expected to improve the performance of its district. This includes optimising the health district as an integrated system and improving the coverage, access, equity and quality of health care and services.

“The purpose of technical support to health districts is to enhance the quality, accessibility, and coverage of health care and services through integrated leadership of the DHMT, which connects health centres and referral hospitals while encouraging the integration of vertical programmes and community participation”. [DR_7_]

In terms of long-term outcomes, an improved population health status is expected. This aligns with the general objective of the DRC’s national health development plan:

“[…] to enhance the overall health of individuals, allowing them to lead a healthy life, and promote well-being for all, regardless of age”. [DR_14_]

#### Intervention

We found that the intervention includes actions at the provincial and district levels.

At the provincial level, actions aim to enhance the abilities of PHA staff to offer effective technical support to DHMT members. These include training sessions and regular meetings of PHA staff.

The training of PHA staff should emphasise action. This aligns with the scoping review’s findings, which identified the ‘action learning or learning-by-doing approach’ [ScR] as a key feature of effective capacity building programmes for district health managers. This is also echoed in the quote below:

“One option to help PHA staff learn their job is to pair them up with an experienced colleague or advisor. This technique of know-how transfer seems the only effective one”. [IDI_2_]

Regular meetings of PHA staff are conversational spaces for PHA staff to discuss technical support issues, share their field experiences and learn from each other:

“Another option [for strengthening the competencies of PHA staff] is to share their experiences. I think there are structures that allow the PHA staff to get together, such as the working groups, the provincial health management team and others. In principle, these meetings should be regular and focus on discussing specific technical support issues, proposing solutions, evaluating their effectiveness, and learning. This may enhance their [PHA staff] competencies through practical experience”. [IDI_2_]

At the district level, technical support for health districts consists of “strengthening the capacities of the DHMT members and healthcare providers […] through training, supportive supervision, problem-solving support, health data analysis, and guidance on health policies and guidelines”. [DR_7_] The terms ‘formative supervision’ and ‘coaching’ are used interchangeably to describe this support. Both focus on “shifting from an administrative and prescriptive approach to a supportive and formative one”. [DR_18_] The key features of this approach are being personalised, problem-solving-centred, reflection-stimulating, regular and continuous and comprehensive.

Personalised support is about providing adequate support for DHMT members, which aligns with their current needs. Involving DHMT members in identifying their own support needs is crucial, as highlighted in the quote below:

“Since coaching is personalised, it should be tailored to the team's or individual's needs; coaching cannot be envisaged on issues decided unilaterally by the coach”. [DR_13_]

Problem-solving support-solving support is an essential component of technical support for DHMT members. PHA staff are expected to have problem-solving skills, as echoed by this informant:

“PHA staff should be able to identify problems, work with DHMT members to find solutions, and provide guidance and adjustments as needed while avoiding taking over the DHMT’s responsibilities”. [IDI_9_]

Reflection-stimulating support enables DHMT members to reflect on and learn from their practices and performances. In such support, the role of the PHA staff in asking the right questions to stimulate reflection within the DHMT is crucial:

“Asking questions, such as ‘why this?’ and ‘why that?’ can help people [DHMT members] reflect on and potentially improve their work. This spirit of reflexivity is often lacking in DHMT members and should be encouraged”. [IDI_8_]

Regular and continuous support is important. Technical support missions should last ‘at least one week’ [IDI_3_] and occur ‘at least once a quarter’. [IDI_6_] The support must be ‘regular and continuous’ [DR_18_] to blend in with the team and ensure a smooth transfer of know-how. Technical support extends beyond field missions, as this informant points out:

“It [technical support] is permanent work with two parts: working in person with frequent and extended visits to the district and working remotely from the PHA office to support the health district. When we were drafting this [PHA] reform, we did not have access to tools like Zoom or WhatsApp, but now we have many more options for improving remote communication during this permanent work”. [IDI_6_]

Comprehensive support: technical support for health districts is intended to cover all aspects of health district development. However, the PHA staff should induce vertical supervision for issues for which they do not have the required skills.

“A PHA staff should take a comprehensive approach to developing the health district to avoid fragmentation. They must also recognise when more than their own skills are needed to meet the needs of DHMT members and bring in additional expertise as needed”. [DR_13_]

#### Context

We organised the contextual factors into the national, provincial and district levels.

At the national level, support from the national MoH is a condition for the success of the provincial-level reform and, thus, technical support for the health districts.

“The success of such restructuring [PHA reform] depends on the support and supervision from the national Ministry of Public Health”. [DR_7_]

The National Health Development Plan 2016–2020 recognised that “the un-reformed national Ministry of Health was not providing enough support for the PHA reform”. [DR1_4_] This is also underlined by one informant in the following terms:

“[...] even at the central level, I get the impression that each of its many departments is working for itself [...], so there is a major lack of harmonisation and coherence in the institutional system that does not make easier the support of the reformed PHA from the central level”. [IDI_2_]

To effectively support PHA, the National Health Development Plan 2016–2020 emphasised the importance of ‘accelerating the reform of the national Ministry of Health’. [DR1_4_]

At the provincial level, we identified two major themes: the optimal functioning of the PHA office and support from the provincial political leaders in the context of decentralisation.

The optimal functioning of the PHA office is a prerequisite for effective technical support to health districts. This implies harmonious coordination among its offices, effective leadership, integration of vertical programmes, availability of resources and an optimal decision-making space.

Harmonious coordination among the various PHA offices is achieved through thematic working groups. These are supposed to facilitate teamwork, break down communication barriers, and enhance participative decision-making and individual and collective learning. They add an adhocratic dimension to PHA functioning.

“It [PHA] is a structure that must stop functioning as a pure administration, leave behind the bureaucratic model and migrate towards a different model, [...] It must function as teamwork without compartmentalisation between offices”. [IDI_5_]

Effective leadership is essential for the optimal functioning of the PHA. This involves a clear and shared vision of the role of the PHA and a ‘willingness to implement reforms’. [DR_12_] Without these, there is a risk of ‘reproducing a dysfunctional system’. [IDI_2_].

“The PHA requires strong leadership [...]. The head of the PHA office should have a clear vision of the expected role of PHA according to the Health System Strengthening Strategy and share it with the staff [...]. This will enable the PHA office to fulfil its mission and play its role more effectively if there is the will”. [IDI_2_]

An optimal level of administrative integration of (vertical) disease control programmes may enable the capture of their financial resources (which ‘account for 60% of financial resources’ [DR_16_]) and better coordinate technical support for health districts, thus preventing overlapping activities.

“The overlap [of technical support] with other activities could be explained by the fact that the funding and actions of specialised programmes still need to be sufficiently integrated at the PHA”. [DR_18_]

Disease control programs rely primarily on external funding, so their integration requires the alignment of funders with national health policies and priorities of PHA. The lack of this alignment may contribute to persistent dysfunction of the health system, as described by this informant:

“Some partners do not align with the national policy or health system strengthening strategy. Due to their financial power, they can influence political decisions and cause disruptions in healthcare organisations. Unfortunately, public funds are limited, and only some activities are funded by those with resources, sometimes at the expense of prioritising the development of health districts in line with the health system strengthening strategy”. [IDI_2_]

Furthermore, the integration of disease control programmes at the national MoH was identified as a prerequisite for their integration at the PHA:

“Integrating specialised programmes at the provincial level may only be successful with national-level reflection and action in this direction”. [DR_19_]

The availability of resources is crucial for the optimal functioning of the PHA office. These resources include first competent and sufficient (number of) human resources to cover all the health districts.

“The PHA must first have the right human resources, i.e. people who are competent and morally upright in sufficient numbers to cover all the health districts”. [IDI_1_]

In addition to the quality and quantity of human resources, adequate financial, material and infrastructural resources are essential to guarantee optimal working and living conditions for PHA staff.

“The biggest issue lies in the precarious living conditions of health workers. Human beings play a crucial role in providing technical support, and to perform their job effectively, they require optimal working and living conditions”. [IDI_2_]

The optimal functioning of the PHA office also requires an optimal decision space for PHA leaders to make the needed decisions and be shielded from harmful influences at the national or provincial level. A participant described the negative influence of national level and political leaders in the following terms:

“They [PHA leaders] do not have the autonomy they should have. They are sometimes influenced by the national level or the provincial authority, which they must continue to satisfy”. [IDI_2_]

Support from the provincial political leaders: In a decentralisation context, certain matters, such as the ‘promotion and organisation of primary health care’ [DR_20_], fall under the exclusive competence of the provinces. Support from provincial political leaders is essential for the success of PHA reform and, thus, technical support for health districts.

“You know, the province has some degree of decentralisation, which means that it has certain responsibilities. Therefore, it is important to have a Governor and a [Provincial] Minister [of Health] who understand and support the PHA in achieving its goals”. [IDI_1_]

The scoping review also identified ‘support from and collaboration with the government authorities’ [ScR] as a factor in the success of capacity building interventions for DHMT members.

At the district level, the contextual factors identified are the leadership and decision space of the DHMT, the working environment and the availability of resources.

The leadership and decision space of DHMT are important for health districts, as noted in the health system strengthening strategy:

“The success of developing health districts relies heavily on the leadership of DHMT. Hence, the DHMT must have a shared vision of the health district's development and the autonomy to make necessary decisions in response to identified problems”. [DR_1_]

According to our scoping review, one of the success factors for capacity building interventions for DHMT members is ‘distributed leadership and the role of the head of health district, who can act as a local champion’. [ScR].

One informant stressed the importance of regular meetings within the DHMT in the following terms:

“It is important to regularly schedule meetings for sharing information and holding each team member accountable for their responsibilities. These meetings also allow the team to acknowledge each other’s contributions and find ways to work together more effectively”. [IDI_5_]

An adequate working environment is essential for the performance of health districts. The informants defined the appropriate working environment as one that ‘offers the right working conditions’ [IDI_2_] and one that ‘offers the necessary resources to carry out the various tasks of the district management team, without outside interferences, especially political interferences’. [IDI_1_] Beyond this material dimension, the scoping review identified the human dimension of the working environment in terms of ‘safe climate work, supportive relationships, teamwork’. [ScR] One informant warns in the following terms:

“The working environment of the DHMT should be noticed. Neglecting to improve it could [negatively] affect the acceptance of technical support and collaboration with PHA staff”. [IDI_4_]

The availability of adequate resources at the district level is crucial for enhancing its performance. These resources include competent human resources and adequate material and financial resources:

“[It is] impossible to develop a health district that is not financed”. [DR_1_]

#### Actors

We identified two categories of key actors: PHA staff and DHMT members.

PHA staff are the providers of technical support to DHMT members. They need professional experience, a gradient of competencies and a positive posture to provide effective technical support to DHMT members.

The PHA staff’s professional experience is important for effective technical support for DHMT members. Indeed, the PHA staff should have ‘successful work experience at the health district level’ [IDI_5_] and ‘useful experience related to the areas they are supporting’ [DR_7_] so that ‘they can use them to support DHMT members in problem-solving’. [IDI_9_] Otherwise, ‘they may only have theoretical knowledge and lack practical reference points’. [IDI_4_].

The PHA staff’s competencies also matter in the technical support process. The PHA staff must have ‘a higher gradient of competencies’ [DR15] than the DHMT members for effective technical support.

“He [PHA staff] must be someone with a higher gradient of competencies than the DHMT members. It would not be appropriate to bring in the health district a PHA staff with a lower level of competencies than the head of the health district or the director of the district hospital, for instance”. [IDI_6_]

We classified the PHA staff's competencies into knowledge, know-how and interpersonal skills. The knowledge and know-how of PHA staff refer to their abilities in management, clinical work and facilitation:

“The PHA staff must have sufficient adequate knowledge of the organisation and functioning of the national health system, policies, strategies and directives, the management of both the health system and health districts, as well as clinical practice”. [DR_7_]

The PHA staff’s interpersonal skills refer to ‘relational qualities made up of a series of attitudes that enable the development of positive and harmonious social relationships’. [DR_12_] The attitudes of good PHA staff that emerged from the interviews and document review include ‘empathy’ [IDI_1_, IDI_6_, DR_7_, DR_12_], ‘listening” [IDI_4_, DR_18_, DR_7_, DR_9_, DR_18_], ‘open-mindedness’ [IDI_1_, IDI_2_, DR_15_], ‘knowing how to communicate or dialogue’ [IDI_1_, IDI_2_, IDI_4_, IDI_5_, DR_7_, DR_9_, DR_15_], ‘observation skills’ [IDI_4_, IDI_8_], ‘humility or modesty’ [IDI_2_, DR_7_], ‘good character or moral probity’ [IDI_1_, DR_7_] and ‘being available’ [IDI_6_, DR_7_, DR_9_, DR_12_].

The PHA staff’s posture is crucial in determining the quality of interactions with DHMT members. Informants agree that PHA staff should avoid being hierarchical and instead adopt a coaching posture to encourage reflection within DHMT.

“In the technical support process, hierarchical posture can bias relationships and hinder the empowerment of individuals and learning processes [...]. Therefore, PHA staff should adopt a professional coach's posture, where they maintain an equal position with the coached team members while still being able to question practices and dynamics. By doing so, the coach becomes an initiator, catalyst, and companion in strengthening the individual and collective competencies, promoting innovation and implementing necessary changes”. [DR_13_]

DHMT members directly benefit from technical support from PHA staff and are key players in developing health districts. DHMTs are ‘interdisciplinary and multiskilled teams’ [DR_4_] and ‘responsible for managing the entire health district’. [DR_1_] The compendium of standards for the organisation and functioning of health districts in the DRC states that the composition of DHMT can vary. However, the members must meet the following criteria: 1) be ‘people capable of working in a team and interested in the dynamic structuring of a health district functioning as an integrated health system’; 2) have ‘a gradient of competencies (acquired through qualification or experience) with staff who are not members of the district management team. Otherwise, supervision is no longer acceptable’; and 3) ‘have broader skills to translate into managerial terms the observations noted in patient management’. [DR_8_].

#### Mechanisms

We identified mechanisms for the PHA staff and DHMT members. Box 2 presents our definitions.

Box 2. Definition of psychological mechanisms identified Self-efficacy refers to the belief of an individual in their ability to perform specific actions that lead to achieving certain goals [37–39].

Motivation is the process through which a person is stimulated to act. Motivation can be extrinsic or intrinsic. Extrinsic motivation is driven by the expectation of receiving a reward or avoiding punishment for performing an activity, while intrinsic motivation stems from an individual’s genuine interest in the activity itself and their ability to derive personal satisfaction from it [40–42].

Psychological safety is a belief shared by individuals about whether it is safe to take interpersonal risks in the workplace [43]. This risk-taking involves speaking up to voice ideas or challenge the status quo without fear of embarrassment, punishment, marginalisation or humiliation [44].

Reflexivity refers to the extent to which a person or a team actively reflects upon their (past) analyses, decisions and actions and how this may lead to adapting them as needed based on current or anticipated circumstances [45, 46].

Trust is a psychological state involving acceptance of vulnerability based on positive expectations of another’s intentions or behaviour [47].

Autonomy is a basic psychological need that means having an optimal degree of freedom and control over one’s actions [40–42].

**Mechanisms for the PHA staff**

Self-efficacy was identified as a key mechanism by PHA staff. They gain skills through training and exchange of experience, which enhance their self-efficacy to effectively provide technical support to health districts:

“The PHA staff member is reassured about his mastery of the issues he discusses with the DHMT members”. [IDI_9_].

Proper preparation of technical visits and continuous self-learning also boost self-efficacy:

“Proper preparation for technical support visits increases the chances of satisfaction for PHA staff in their coaching role. This is because good results improve self-efficacy and motivate individuals to move forward”. [DR_13_]

Motivation was found to be a key mechanism for providing effective technical support to DHMT members. Respondents state that this motivation is extrinsic, i.e. linked to financial incentives and to the working environment and conditions:

“It is clear that the motivation of health workers in the resources-limited context is a delicate issue. However, each PHA office in the country should have minimum funding (considering state wages, risk premiums, and funds from other partners) that needs to be coordinated for adequate technical support to health districts [...]. Boosting the PHA staff's motivation does not solely rely on financial incentives. It also involves enhancing the working environment, teamwork, relationships between colleagues, and the leadership quality of the head of PHA. Recognising and appreciating small achievements and providing opportunities for PHA staff to upgrade their skills can increase the PHA staff's motivation even in a challenging environment”. [IDI_5_]

Reflexivity was found to be helpful for individual and collective learning. The interviews and the document review showed that PHA staff should be reflexive and instil this attitude in DHMT members.

“The preparatory and debriefing meetings for technical support visits at the PHA office are opportunities for PHA staff to question their practices, share their experiences and learn from each other”. [IDI_9_]

“The PHA staff should develop methods that encourage supported teams to question their performance, practices, working methods regarding the problems that arise, the health district's objectives and their working environment”. [DR_13_]

Psychological safety was also found to be a key mechanism for individual and collective learning. It depends on the leadership of the head of the PHA office.

“The PHA’s activities are coordinated through regular meetings to discuss necessary actions in response to identified problems. The preparation, debriefing of technical missions and analysis of health district data are suitable opportunities for these discussions. The head of the PHA office should create the right conditions for open and cordial discussions to take place, leading to consensus-based decisions”. [IDI_9_]

**Mechanisms for DHMT members**

Perceived credibility of the PHA staff: DHMT members are more likely to accept and actively participate in the technical support process when they perceive their coach as credible. This perception “is nourished by the knowledge, know-how and interpersonal skills of the PHA staff […] and is necessary for him to have a certain leadership, recognition and influence over the DHMT members”. [DR_12_] This is also explained in the following excerpt:

“The PHA staff improves his or her own knowledge and skills, which enables him or her not to lose face in front of the teams being supported and thus maintain credibility and legitimacy in their eyes”. [DR_13_]

Perceived relevance of the support: DHMT members are more likely to accept and actively participate in the technical support process when they perceive it as relevant. This positive perception is triggered if “the support is consistent with the legitimate needs of those being supported”. [DR_13_].

“The management teams in the district are looking for support that genuinely meets their expressed needs. It is essential for the support provided by the PHA staff to align with the actual needs identified at the health district level”. [IDI_5_]

The scoping review stressed the importance of tailoring capacity building programmes to the needs of district health managers.

“[…] adaptability and flexibility of CBP [capacity building programme] processes make them more responsive as they consider the needs of DHMs [district health managers] and their context, which contribute to increased perceived relevance and sense of ownership by DHMs”. [ScR]

Trust in the PHA staff: supportive relationships foster trust in the PHA staff by DHMT members, which promotes openness and willingness to learn and embrace change:

“Establishing trust is crucial for effective knowledge transfer and district management team members' acceptance of the supervisor’s feedback”. [IDI_9_]

“The supervisor’s attitude will impact the level of trust among the supervised [district management] team members. This will influence their ability to confide in each other and engage in honest and productive dialogue”. [DR_15_]

The mutual trust between facilitators and participants has been identified in the scoping review as a key driver of participation in the capacity building programme for district health managers.

“[…] supportive interactions between facilitators and DHMs [district health managers], which enable mutual trust and enhance motivation and commitment of DHMs to actively participate in the CBP [capacity building programme] process and to engage with changes”. [ScR]

Self-efficacy: technical support as a capacity-building intervention enhances district management team members’ knowledge and practical skills, which in turn trigger their can-do attitude when carrying out their managerial or leadership tasks at the health district level.

“Capacity building programmes methods, such as team-based training, learning-by-doing approach, a shift from administrative and control to a supporting model of supervision, reflective discussions for continuous learning […] empower DHMs and activate a can-do attitude (self-efficacy)”. [ScR]

Perceived autonomy: effective technical support combined with optimal decision spaces increases the autonomy of DHMT members in performing their duties. The perceived autonomy of DHMTs combined with self-efficacy can increase motivation and improve the team’s performance.

“Effective coaching enhances the autonomy of individuals or teams being coached and continuously improves the team's performance”. [DR_13_]

Psychological safety was also found to be a key mechanism for learning at the interface between the PHA staff and DHMT members. It depends on the interpersonal skills and posture of PHA staff:

“As you know, technical support is a learning process. It is not a unidirectional but rather a bidirectional process. The DHMT members learn from the PHA staff, and the PHA staff also learn from them. To learn, PHA staff should be humble and know how to communicate and listen effectively. Listening requires giving DHMT members the chance to ask questions and voice their opinions. […] The PHA staff should avoid a hierarchical approach by assuming they are superior and can dictate what DHMT members should do. There needs to be an exchange of ideas; otherwise, the DHMT members will become frustrated and reluctant to share their ideas”. [IDI_2_]

### ICAMO configurations

We identified six ICAMO configurations, two at the provincial level, three at the interface between PHA staff and DHMT members and one at the health district level (Table 3).

**Table 3:** ICAMO configurations

Intervention	Context	Actors	Mechanisms	Outcomes
At the PHA level				
1. Adequate training of PHA staff	Supportive leadership (good work climate and promotion of positive values) Availability of adequate resources	PHA staff	Self-efficacy – motivation	Improved competencies of PHA staff
2. Regular meetings for technical support visits	Supportive leadership Safe conversational space Less hierarchical management culture	PHA staff	Psychological safety Reflexivity	Improved competencies through individual and collective learning
At the interface between PHA staff and DHMT members				
3. Needs-driven or personalised support	Enabling learning environment (judgement-free, fault-accepting, non-threatening and less hierarchical) Optimal integration of vertical programmes	DHMT members	Positive perceived support’s relevance	Improved competences through active participation
4. Problem-solving – centred support	Enabling learning environment (judgement-free, fault-accepting, non-threatening and less hierarchical) Competent PHA staff (good management, facilitation and relational skills)	DHMT members	Positive perceived credibility of PHA staff Trust in PHA staff	Improved competences through active participation
5. Reflection – stimulating support	Enabling learning environment (judgement-free, fault-accepting, non-threatening and less hierarchical) Competent PHA staff (good management, facilitation and relational skills)	DHMT members	Psychological safety Reflexivity	Improved competences through individual and collective learning
At the district level				
6. Good management practices at district level	Supportive leadership, optimal decision space, supportive work conditions, availability of resources and absence of negative political influences	DHMT members	Self-efficacy—perceived autonomy	Improved performance of health districts

#### ICAMO configuration 1

Training in management and facilitation, including relational knowledge and skills (I) targeting PHA staff (A), increases their self-efficacy (M) and motivation (M), leading to improved competencies (O) and commitment to providing technical support to DHMT members (O). A good work climate, promotion of positive values and provision of adequate resources (C) at the PHA office are essential.

Unsupportive leadership in a context of inadequate resources (C) may demotivate (M) PHA staff (A) and lead to the exit of staff, reducing the number of skilled staff at the PHA office and thus jeopardising the technical support process (O).

#### ICAMO configuration 2

Regular meetings at the PHA office to plan, evaluate and discuss technical support issues (I) offer PHA staff (A) opportunities to share, reflect on and learn from their field experiences, enabling psychological safety (M) among PHA cadres and contributing to reflexivity (M), which leads to improved competencies (O) through individual and collective learning on the condition of safe conversational spaces that values and respects everyone’s opinions and encourages people to speak up (C).

Conversely, a highly hierarchical management culture (C) can create psychological unsafety (M), making PHA staff (A) hesitant to share their opinions for fear of being judged, embarrassed or punished. This inhibits both reflexivity and learning and thus hinders the development of competencies of PHA staff (O).

#### ICAMO configuration 3

DHMT members’ involvement in identifying their own support needs and planning support visits (I) results in a positive perception of the relevance of the support received (M), encouraging their active participation in the technical support process and improving their competencies (O). This occurs more likely in an environment conducive to learning (i.e. that is judgement-free, fault-accepting, non-threatening and less hierarchical and where there are supportive relationships between PHA staff and DHMT members) (C).

Conversely, vertical supervision visits by disease control programme staff (I) that do not necessarily meet the needs of DHMT members (A), lead to perceptions of irrelevance of such supervision (M), hindering their professional development and ultimately resulting in less than optimal performance (O).

#### ICAMO configuration 4

DHMT members (A) are likely to participate effectively in technical support and thus improve their competencies (O) if they perceive the PHA staff as credible (M) and trustworthy (M). These positive perceptions of credibility and trustworthiness are triggered if the PHA staff has good management, facilitation and relational skills (A), which allow them to provide effective problem-solving support (I) to DHMT members and set up a conducive learning environment that is judgement-free, fault-accepting, non-threatening and less hierarchical and fosters supportive relationships with DHMT members (C).

However, supervisions by PHA staff members with a hierarchical attitude (I) may be perceived as less credible (M) and trustworthy (M) by the DHMT members (A), hinder their psychological safety (M) and result in weak or reluctant participation in the technical support process (O), ultimately hampering the performance of health districts (O).

#### ICAMO configuration 5

If competent PHA staff members stimulate meaningful reflections and provide constructive feedback (I), in a learning environment that is judgement-free, fault-accepting, non-threatening and non-hierarchical, and where relationships between PHA staff and DHMT members are supportive (C), then DHMT members may become more reflexive (M), which contributes to individual and collective learning and ultimately improved competencies (O).

#### ICAMO configuration 6

If supervision (I) increases their competences, DHMT members (A) will be more motivated to develop management initiatives to improve their health districts’ performance (O) because of higher self-efficacy (M) and perceived autonomy (M). Favourable contextual conditions include strong leadership, a supportive work environment with adequate resources and an absence of negative political influences (C).

### The initial programme theory

On the basis of the ICAMO configurations, we drafted an initial programme theory:

Adequate training of PHA staff that is based on an action-learning approach and regular meetings to plan, evaluate and discuss technical support for DHMT members improves their competencies by increasing self-efficacy, motivation, psychological safety and reflexivity. Effective leadership, availability of resources and a safe conversational space at the PHA office are crucial context factors.

At the interface between PHA staff and DHMT members, technical support for DHMT members that addresses their needs and provides effective problem-solving, meaningful reflections and constructive feedback can trigger positive perceived relevance of support, positive perceived credibility of PHA staff, trust in PHA staff and psychological safety. This, in turn, can lead to improved competencies of DHMT members through their active participation in technical support processes and individual and collective learning. It requires a conducive environment for learning (judgment-free, accepting faults, non-threatening and non-hierarchical), optimal integration of vertical-specific disease programmes and competent PHA staff with good management, facilitation and relational skills.

At the district level, effective technical support increases the competencies, self-efficacy and perceived autonomy of DHMT members, who will more easily develop initiatives to improve their performance. Favourable contextual conditions include effective leadership, a supportive work environment, adequate resources and an absence of negative political influences.

**Fig. 3:**
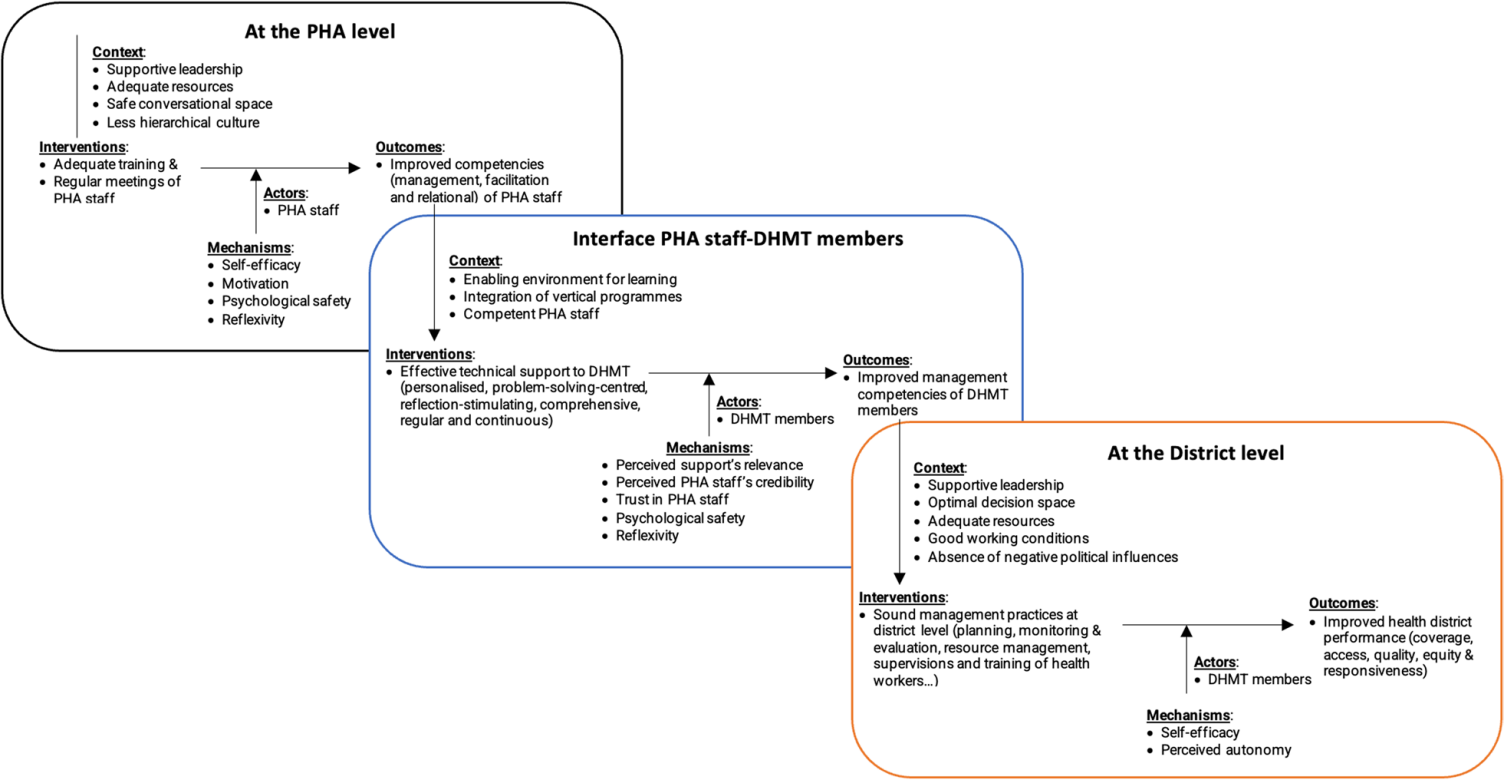
The initial programme theory

In summary, the IPT is outlined in Fig. 3

## Discussion

In this paper, we identify ICAMO components and formulate six ICAMO configurations explaining how technical support processes bring about expected outcomes by triggering mechanisms for PHA staff and DHMT members under specific contextual conditions.

The IPT emphasises the importance of an action-learning approach at both the provincial and district levels. Action-learning focuses on action (or experience) and reflection as sources of learning. Indeed, intervention components at the provincial level (PHA staff training and meetings) involve action and reflection. Similarly, key features of effective technical support for DHMT members identified in this study (personalised, problem-solving-centred and reflection-stimulating) involve action and reflection. The action-learning approach is rooted in adult learning theories, which are Kolb’s experiential learning theory [48, 49], Knowles’ adult learning theory [50] and Mezirow’s transformative learning theory [51].

In Kolb’s experiential learning theory, the learning cycle consists of four stages: concrete experience, reflective observation, abstract conceptualisation and active experimentation. This implies that concrete experiences lead to reflective observation, from which abstract concepts are developed and tested in new experiences [48, 49]. When applied to the technical support process, PHA staff and DHMT members can learn from their practical experiences by reflecting on them. Knowles’ adult learning theory highlights the importance of self-directed learning, experiences (including errors), perceived relevance, problem-solving and intrinsic motivation in the learning process [50]. By involving DHMT members in the planning and evaluation of their own learning experiences and tailoring technical support approaches to their unique needs and experiences, a learner-centred environment can be created. Mezirow’s transformative learning theory suggests that learning occurs when people critically reflect on their values, beliefs and assumptions, leading to new and meaningful perspectives. This theory emphasises six key elements: individual experience, critical reflection, dialogue, holistic orientation, awareness of context and authentic relationships [51]. PHA can facilitate transformative discussions that help DHMT members question and reframe their perspectives. From the preceding, it is evident that the three adult learning theories encompass key technical support features (personalised, problem-solving-centred and reflection-stimulating support). Integrating these adult learning theories within the technical support process may enhance its effectiveness by promoting experiential, transformative and self-directed learning tailored to the unique context of DHMT members. Additionally, these theories highlight the importance of perceived relevance in the learning process.

This perceived relevance can be linked to the health belief model [52] and to the integrated theory of health behaviour change [53]. The health belief model suggests that the perceived benefits or positive consequences of a health behaviour can influence its adoption [52]. According to the integrated theory of health behaviour change, enhancing personal perceptions (such as self-efficacy, outcomes expectancy and goal congruence) and social support (emotional, instrumental or informational) can lead to engagement in a health behaviour [53]. Similarly, DHMT members are more likely to accept and actively participate in the technical support process if they perceive it as relevant to their needs and expectations (outcomes expectancy and goal congruence). This perception of relevance is a crucial mechanism in their engagement because they may believe that the technical support will equip them with new knowledge and skills (benefits) they can use to enhance their professional growth. When technical support is aligned with their professional needs and challenges, it becomes more effective and meaningful.

Effective technical support involves supportive relationships between PHA staff and DHMT members and can enhance the motivation, self-efficacy and autonomy of DHMT members as well as PHA staff. These mechanisms refer to self-determination theory [40], according to which every person seeks to fulfil three fundamental psychological needs – autonomy, competence and relatedness – which are essential for their optimal motivation, engagement and well-being [40, 42]. Competence involves feeling effective or having self-efficacy when performing work tasks. Bandura [37] proposed four sources of self-efficacy: mastery experiences, vicarious experiences, verbal persuasion and physiological and affective states. During the technical support process, PHA staff can boost the self-efficacy of DHMT members by leveraging these four sources. First, they can encourage the implementation of sound management practices and skill development to give DHMT members positive mastery experiences. Second, they can act as role models or share success stories from other DHMTs to enhance the belief of DHMT members that they too can perform effectively in their roles (vicarious experiences). Third, PHA staff can provide positive feedback and express confidence in the capabilities of DHMT members, thereby increasing their self-efficacy through social persuasion. Finally, a supportive and positive emotional environment can be fostered by the PHA staff during the technical support process in order to reduce stress and enhance overall well-being, leading to a heightened sense of self-efficacy among DHMT members (affective states). By systematically reinforcing each of these four sources of self-efficacy, PHA staff can create a learning environment that empowers DHMT members and builds and sustains their confidence in their abilities. This learning environment can foster relatedness or a sense of belonging to a social group [40, 42, 54], which can lead to psychological safety [55]. Enhanced self-efficacy can lead to autonomy, allowing DHMT members to have freedom and control over their actions.

Psychological safety was found to be a mediator between antecedents such as supportive leadership behaviour, supportive organisational practices and relationship networks and positive work outcomes such as team learning, performance, innovation [45, 56] and reflexivity [46]. Team learning – known as a continuous process of questioning, reflecting, experimenting, seeking feedback and discussing outcomes or errors [43] – is linked to reflexivity. The level of reflexivity within a team is influenced by various factors, such as the level of trust and psychological safety among team members, a shared vision, diversity and leadership style. Higher levels of reflexivity can lead to increased innovation, effectiveness, and creativity within a team [46, 56–58]. Psychological safety is related to trust, another key mechanism for the learning process. Both psychological safety and trust refer to the climate within a team regarding the expectation of cooperative or non-harming behaviour of the PHA staff or other DHMT members [46, 59–61]. Trust is essential in daily workplace dynamics and fosters positive relationships. It has been linked to the improved intrinsic motivation of health workers [62], improved team performance and positive work outcomes, such as better organisational citizenship behaviour [63]. The relational skills of PHA staff are incredibly important for activating these mechanisms. In fact, PHA staff must adopt a suitable attitude that promotes reflection, ensures psychological safety and instils trust to facilitate an effective learning process. Fostering psychological safety within a team creates an environment where team members feel comfortable expressing themselves, taking risks, and learning from experiences. This, in turn, enhances reflexivity – individual and collective reflection – which may in turn lead to increased innovation and effectiveness as the team continually adapts, learns and generates creative solutions to challenges.

Our findings indicate that technical support is a multi-level process operating at the PHA office, the PHA staff-DHMT members interface and the health district levels. Figure 2 illustrates the connection between these levels, showing the influence of the higher level’s outcomes on the immediate lower level’s context. This phenomenon is referred to as the ‘ripple effect’ in realist literature [64]. It is a consequence of complex interdependencies within the health system, requiring better coordination of activities across levels to ensure that any change in one level does not negatively affect other levels [65].

### Rigour and trustworthiness

In the realist approach, the rigour of a study depends on ‘the trustworthiness of the evidence source and the coherence of programme theory’ [66]. To ensure the trustworthiness of our study findings, we gathered data from primary and secondary sources (interviews with programme designers, scoping review and document review) and triangulated them during data analysis. Triangulation involves comparing and contrasting information from multiple perspectives to gain a more comprehensive understanding of the research phenomenon [67]. By cross-verifying data from different sources and methods, triangulation helped us to minimise the bias associated with a single method or data source, and this increased the reliability, credibility and validity of our findings. Our analysis went beyond the mere thematic categorisation of intervention features, context, actors, mechanisms and outcomes (ICAMO components) to delve deeper into the relationships between them (i.e. developing ICAMO configurations) [68].

We enhanced the coherence of our programme theory in various ways. First, we searched for rival theories, i.e. alternative statements hypothesising how the same programme resources could result in different responses and outcomes [69]. Second, we discussed the articulation of our programme theory with relevant substantive theories, such as adult learning theories (including Kolb’s experiential learning theory, Knowles’ adult learning theory and Mezirow’s transformative learning theory), health behaviour change theories (such as the health belief model and the integrated theory of health behaviour change) and self-determination theory [66, 68]. These theories complement each other in explaining the learning process. Indeed, most adult learning theories underscore the significance of self-directed learning and the real-life application of knowledge. They are aligned with the principles of self-determination theory that emphasise autonomy in the learning process. Health behaviour change theories offer insights into the factors influencing DHMT members’ behaviour and motivation. Integrating these theories with adult learning principles may enable PHA staff to tailor technical support to address the specific needs, challenges and motivations of DHMT members. Self-determination theory, according to which intrinsic motivation is the result of fulfilling the psychological needs of autonomy, competence and relatedness, can be combined with adult learning and health behaviour theories. However, the integration of multiple theories is a complex process, requiring a nuanced understanding of each theory’s principles. To facilitate this integration, it is essential to clearly define the connections between theories and provide practical guidelines for PHA staff to apply them cohesively in supporting DHMT members.

Finally, we discussed and sought feedback from supervisors who are experts in realist evaluation and health systems and policy research and are well-versed in the Congolese health system. Such discussions and feedback ensured the appropriateness of methods, data analysis and interpretations and contributed to the credibility of our study. Furthermore, we adhered to the reporting standards for realist evaluation (RAMESES II checklist, Additional file 3) [70] and qualitative research (COREQ checklist, Additional file 4) [71]. These standards are designed to improve the comprehensiveness, consistency and rigour of research reporting. In addition, they enhance transparency, facilitate reproducibility and enable quality assessment of studies. They help readers to better understand the design, conduct, analysis and findings of the studies [70–73]. However, reporting standards have some limitations. One of the limitations is that they primarily address the reporting phase of research, emphasizing how research is communicated rather than how it is conducted. While transparent reporting is crucial, it does not guarantee that there are no methodological flaws or biases in the actual research process [72]. To minimize this limitation, we combined the use of these checklists with the quality standards for realist evaluation for evaluators and peer-reviewers set by the RAMESES II Project [74].

### Strengths and limitations of the study

A key strength of this study is the use of multiple strategies (data source triangulation, search for rival theories, articulation with relevant substantive theories, expert audit and adherence to reporting standards) to ensure the rigour (trustworthiness and coherence) of our study.

However, this study has some limitations. First, the insights from the scoping review were helpful in developing the IPT. However, it is important to note that only a few studies were conducted in fragile settings like the DRC. This raises questions about the necessary contextual factors that are needed for effective capacity development processes. Second, we presented the ICAMO configurations discretely and linearly. In reality, we expect the underlying pathways to be more intricate and interdependent. Technical support for DHMT members is, indeed, a complex capacity-building intervention that a configurational analysis cannot fully describe. Pawson and Tilley advise realist researchers to acknowledge the complexity of their subject matter and remain humble in their approach, recognising that their ‘understanding will always be partial and provisional’ [20]. Realist inquiry outputs are approximate in nature, which requires them to accumulate over time through recurrent theory testing and refinement cycles [26, 28]. Third, it is worth noting the possibility of recall bias, particularly for the designers of the intervention which is over 5 years old, and the social desirability bias during this study. We took measures to minimize these biases by using triangulation of data sources and methods [30].

## Conclusions

Technical support for DHMTs is a complex intervention in a complex health system. This study allowed us to identify six ICAMO configurations that explain how effective technical support, which is personalised, problem-solving-centred and reflection-stimulating, can improve the competencies of DHMT members. This improvement can be achieved through the activation of various mechanisms, such as the positive perceived relevance of the support, positive perceived credibility of PHA staff, trust in PHA staff, psychological safety, reflexivity, self-efficacy and perceived autonomy. These mechanisms operate within specific contextual conditions, including an enabling learning environment, the integration of vertical programs, competent PHA staff, optimal decision-making space, supportive work conditions, availability of resources and the absence of negative political influences. These ICAMO configurations will be tested in subsequent empirical studies.

### Supplementary Information

**Additional file 1.** Description of documents included in the review document.

**Additional file 2.** Interview guide for programme designers.

**Additional file 3.** Checklist for realist evaluation studies.

**Additional file 4.** COREQ checklist.

## Data Availability

The dataset supporting the conclusions of this article is included in the article (and its supplemental files).

## Abbreviations

DRC: Democratic Republic of Congo
DHMT: District health management team
HSSS: Health system strengthening strategy
ICAMO: Intervention–context–actor–mechanism–outcomes
IPT: Initial programme theory
MoH: Ministry of Health
PHA: Provincial Health Administration
RE: Realist evaluation

## References

[1] Meessen B, Van Damme W (2005). Systèmes de santé des pays à faible revenu: Vers une révision des configurations institutionnelles?. Mondes Dev.

[2] Alliance for Health Policy and Systems Research. Strengthening health systems: the role and promise of policy and systems research. Geneva; 2004.

[3] Papanicolas I, Rajan D, Karanikolos M, Soucat A, Soucat A (2022). Health system performance assessment. A framework for policy analysis.

[4] Senkubuge F, Modisenyane M, Bishaw T (2014). Strengthening health systems by health sector reforms. Glob Health Action.

[5] Bossert T (1998). Analyzing the decentralization of health systems in developing countries: Decision space, innovation and performance. Soc Sci Med.

[6] World Health Organization. The World health report 2000: health systems: improving performance [Internet]. Geneva: World Health Organization; 2000. https://apps.who.int/iris/handle/10665/42281

[7] World Health Organization. Report on the performance of health systems in the WHO Africa Region. 2020.

[8] Fullman N, Yearwood J, Abay SM, Abbafati C, Abd-Allah F, Abdela J (2018). Measuring performance on the Healthcare Access and Quality Index for 195 countries and territories and selected subnational locations: a systematic analysis from the Global Burden of Disease Study 2016. Lancet.

[9] Kalambay H, Wim N, Lerberghe V. Improving health system efficiency: Democratic Republic of the Congo: improving aid coordination in the health sector. 2015. https://apps.who.int/iris/handle/10665/186673. Accessed 17 May 2022.

[10] Ministère de la Santé. Stratégie de Renforcement du Système de Santé. République Démocratique du Congo; 2010.

[11] Ministère de la Santé. Stratégie de Renforcement du Système de Santé. République Démocratique du Congo; 2006.

[12] Bosongo SI, Mukalenge FC, Tambwe AM, Criel B (2021). Les médecins prestataires à la première ligne des soins dans la ville de Kisangani en République Démocratique du Congo: vers une typologie. Afr J Prim Health Care Fam Med.

[13] Présidence de la République. Constitution de la République Démocratique Du Congo. Journal Officiel de la République Démocratique du Congo. 2006;

[14] Kahindo M, Schirvel C, Godelet E, Wodon A, Porignon D, Bonami M (2014). Réforme des structures intermédiaires de santé en République démocratique du Congo. Sante Publique.

[15] Ministère de la Santé Publique RDC. Référentiel de compétences intégré de l’équipe cadre de la zone de santé. Pour les situations professionnelles relatives au management de la zone de santé en RDC. Kinshasa; 2010.

[16] Kahindo MJB, Mitangala NP, Tsongo ME, Mahamba N, Kahandukya NL, Kubuya Bonane J (2021). Soutien du niveau intermédiaire du système au district de santé: Perceptions des équipes de district de santé du Nord Kivu à l’est de la RDC. Int J Innov Appl Stud.

[17] Chuy KD, Criel B, Belrhiti Z, Mwembo TA, Chenge MF (2020). Soutien du niveau intermédiaire du système de santé aux équipes cadres des districts sanitaires: Le cas de la ville de Lubumbashi, République Démocratique du Congo. Int J Adv Res (Indore).

[18] Prashanth NS, Marchal B, Devadasan N, Kegels G, Criel B (2014). Advancing the application of systems thinking in health: a realist evaluation of a capacity building programme for district managers in Tumkur, India. Health Res Policy Syst..

[19] Pawson R, Tilley N (1997). Realistic evaluation.

[20] Pawson R, Tilley N, Pawson R, Tilley N. Realist Evaluation Realist Evaluation. 2004; http://www.communitymatters.com.au/RE_chapter.pdf

[21] Dalkin SM, Greenhalgh J, Jones D, Cunningham B, Lhussier M (2015). What’s in a mechanism? Development of a key concept in realist evaluation. Implement Sci.

[22] Lacouture A, Breton E, Guichard A, Ridde V (2015). The concept of mechanism from a realist approach: a scoping review to facilitate its operationalization in public health program evaluation. Implement Sci.

[23] Lemire S, Kwako A, Nielsen SB, Christie CA, Donaldson SI, Leeuw FL. What is this thing called a mechanism? Findings from a review of realist evaluations. In: Causal Mechanism in Evaluation. New Direct. 2020.

[24] Smeets RGM, Hertroijs DFL, Mukumbang FC, Kroese MEQL, Ruwaard D, Elissen AMJ (2022). First things first: how to elicit the initial program theory for a realist evaluation of complex integrated care programs. Milbank Q.

[25] Mukumbang FC, Kabongo EM, Eastwood JG (2021). Examining the application of retroductive theorizing in realist-informed studies. Int J Qual Methods.

[26] Jagosh J (2020). Retroductive theorizing in Pawson and Tilley’s applied scientific realism. J Crit Realism.

[27] Merton RK (1968). Social Theory and Social Structure.

[28] Marchal B, van Belle S, van Olmen J, Hoerée T, Kegels G (2012). Is realist evaluation keeping its promise? A review of published empirical studies in the field of health systems research. Evaluation.

[29] Marchal B, Kegels G, Van Belle S. Theory and realist methods. In: Emmel N, Greenhalgh J, Manzano A, Monaghan M, Dalkin S, editors. Doing realist research. 23th edition. 2017.

[30] Mukumbang FC. A realist evaluation of the antiretroviral treatment adherence club programme in the metropolitan area of the Western Cape Province, South Africa. University of the Western Cape; 2018. http://etd.uwc.ac.za 10.1136/bmjopen-2015-009977 PMC4823437 27044575

[31] Bosongo S, Belrhiti Z, Ekofo J, Kabanga C, Chenge F, Criel B (2023). How capacity building of district health managers has been designed, delivered and evaluated in sub-Saharan Africa: a scoping review and best fit framework analysis. BMJ Open.

[32] Mukumbang FC, Van Belle S, Marchal B, Van Wyk B (2016). Towards developing an initial programme theory: Programme designers and managers assumptions on the antiretroviral treatment adherence club programme in primary health care facilities in the metropolitan area of western cape province, South Africa. PLoS ONE.

[33] Bowen GA (2009). Document analysis as a qualitative research method. Qual Res J.

[34] Ritchie J, Spencer L, Bryman A, Burgess B (1994). Qualitative data analysis for applied policy research. Analyzing qualitative data.

[35] Kleinheksel AJ, Rockich-Winston N, Tawfik H, Wyatt TR (2020). Qualitative research in pharmacy education. Demystifying content analysis. Am J Pharm Educ.

[36] Mukumbang FC, Marchal B, Van Belle S, van Wyk B (2020). Using the realist interview approach to maintain theoretical awareness in realist studies. Qual Res.

[37] Bandura A (1997). Self-efficacy: the exercise of control.

[38] Bandura A (1986). Social foundations of thought and action: a social cognitive theory.

[39] Bandura A (1977). Self-efficacy: toward a unifying theory of behavioral change. Psychol Rev.

[40] Ryan RM, Deci EL (2000). Self-determination theory and the facilitation of intrinsic motivation. Soc Dev Well-Being American Psychol.

[41] Deci EL, Ryan RM. Self-Determination Theory. In: International Encyclopedia of the Social & Behavioral Sciences: Second Edition. Elsevier Inc.; 2015. p. 486–91.

[42] Legault L. Self-Determination Theory. In: Encyclopedia of Personality and Individual Differences. Cham: Springer International Publishing; 2017. p. 1–9. 10.1007/978-3-319-28099-8_1162-1

[43] Edmondson A (1999). Psychological safety and learning behavior in teams. Adm Sci Q.

[44] Edmondson AC, Lei Z (2014). Psychological safety: the history, renaissance, and future of an interpersonal construct. Annu Rev Organ Psych Organ Behav.

[45] West MA. View graph of relations Reflexivity, revolution, and innovation in work teams. Beyerlein M, Johnson D, Beyerlein S, editors. Stamford: JAI Press; 2000. 1–29 p.

[46] Widmer PS, Schippers MC, West MA (2009). Recent developments in reflexivity research: a review. Psychol Everyday Activity..

[47] Rousseau DM, Sitkin SB, Burt RS, Camerer C (1998). Not so different after all: a cross-discipline view of trust. Acad Manag Rev.

[48] Kolb DA, Boyatzis RE, Mainemelis C, Sternberg RJ, Zhang LF. Experiential learning theory: previous research and New Directions. 2000.

[49] Kolb AY, Kolb DA. Experiential learning theory: A dynamic, holistic approach to management learning, education and development. In: The SAGE Handbook of Management Learning, Education and Development. SAGE Publications Inc.; 2009. p. 42–68.

[50] Knowles MS, Holton EF, Swanson RA (2005). The adult learner: the definitive classic in adult education and human resource development.

[51] Mezirow J (1991). Transformative dimensions of adult learning.

[52] Green EC, Murphy EM, Gryboski K. The Health Belief Model. In: The Wiley Encyclopedia of Health Psychology. 2020, 211–4. 10.1002/9781119057840.ch68

[53] Ryan P (2009). Integrated theory of health behavior change: background and intervention development. Clin Nurse Spec.

[54] Belrhiti Z, Van Damme W, Belalia A, Marchal B (2020). Unravelling the role of leadership in motivation of health workers in a Moroccan public hospital: a realist evaluation. BMJ Open.

[55] Aboud K, Xiongying N, Qasim M (2023). Impact of ethical leadership on employees’ psychological safety and voice behavior; with mediating role of belongingness. Int J Sci Bus..

[56] Carter SM, West MA (1998). Reflexivity, effectiveness and mental health in BBC-TV production teams. Small Group Res.

[57] Schippers MC, West MA, Edmondson AC. Team Reflexivity and Innovation. In: The Wiley Blackwell Handbook of the Psychology of Team Working and Collaborative Processes. Wiley; 2017. p. 459–78.

[58] Tjosvold D, Tang MML, West M (2004). Reflexivity for team innovation in China: the contribution of goal interdependence. Group Organ Manag.

[59] Gilson L (2006). Trust in health care: theoretical perspectives and research needs. J Heal Organ Manag.

[60] Gilson L (2003). Trust and the development of health care as a social institution. Soc Sci Med.

[61] Goudge J, Gilson L (2005). How can trust be investigated? Drawing lessons from past experience. Soc Sci Med.

[62] Okello DRO, Gilson L (2015). Exploring the influence of trust relationships on motivation in the health sector: a systematic review. Hum Resour Health.

[63] Simpson JA, Vieth G, Krueger F (2021). Trust and psychology: psychological theories and principles underlying interpersonal trust. The neurobiology of trust.

[64] Jagosh J, Bush PL, Salsberg J, Macaulay AC, Greenhalgh T, Wong G (2015). A realist evaluation of community-based participatory research: partnership synergy, trust building and related ripple effects. BMC Public Health.

[65] Trickett EJ, Beehler S (2013). The ecology of multilevel interventions to reduce social inequalities in health. Am Behav Sci.

[66] Dada S, Dalkin S, Gilmore B, Hunter R, Mukumbang FC (2023). Applying and reporting relevance, richness and rigour in realist evidence appraisals: advancing key concepts in realist reviews. Res Synth Methods.

[67] Patton MQ (2002). Qualitative evaluation and research methods.

[68] The RAMESES II Project. Quality standards for realist evaluation: for evaluators and peer-reviewers. 2017. www.ramesesproject.org. Accessed 8 Sep 2023.

[69] Jagosh J, Stott H, Halls S, Thomas R, Liddiard C, Cupples M (2022). Benefits of realist evaluation for rapidly changing health service delivery. BMJ Open.

[70] Wong G, Westhorp G, Manzano A, Greenhalgh J, Jagosh J, Greenhalgh T (2016). RAMESES II reporting standards for realist evaluations. BMC Med.

[71] Tong A, Sainsbury P, Craig J (2007). Consolidated criteria for reporting qualitative research (COREQ): a 32-item checklist for interviews and focus groups. Int J Qual Health Care.

[72] Wharton T (2017). Rigor, transparency, and reporting social science research: why guidelines don’t have to kill your story. Res Soc Work Pract.

[73] McLeroy KR, Garney W, Mayo-Wilson E, Grant S (2016). Scientific reporting: raising the standards. Health Educ Behav.

[74] The RAMESES II Project. Quality standards for realist evaluation. For evaluators and peer-reviewers. 2017. www.ramesesproject.org

