# Supplementary material for: The role of provincial health administration in supporting district health management teams in the Democratic Republic of Congo: eliciting an initial programme theory of a realist evaluation
Source: Health Res Policy Syst. 2024 Feb 20;22:29. doi: 10.1186/s12961-024-01115-9 (PMC10880215; doi:10.1186/s12961-024-01115-9)
Supplement: Supplementary file 1 — Additional file 1. Description of documents included in the review document. [file 12961_2024_1115_MOESM1_ESM.docx]

Additional file 1. Description of included documents in the review document.

| **#** | **Title** | **Author/Year** | **Document type** | **Description** |
| --- | --- | --- | --- | --- |
| 1 | Stratégie de Renforcement du Système de Santé - 1ère édition | Ministère de la Santé Publique - RDC (2006) | Health Policy document | This document established a coherent diagnosis of the DRC's health system weaknesses. It proposed a strengthening strategy that emphasises the development of health districts and calls for reforming national and provincial health administrations. |
| 2 | Recueil des normes de la zone de santé | Ministère de la Santé Publique - RDC (2006) | Health Policy document | This document is a collection of norms for the organisation and functioning of health districts in the DRC. In particular, it describes the mission, functions and composition of health district management teams as well as the profile of their members. |
| 3 | Stratégie de Renforcement du Système de Santé - 2ème édition | Ministère de la Santé Publique - RDC (2010) | Health Policy document | This document is an update of the first edition (document #1). |
| 4 | Référentiel des Compétences Intégrées de l'équipe cadre de zone de santé | Parent F. et al. (2010) | Health Policy document | This document outlines the key managerial competencies, skills and situations that a district health management team should develop in order to carry out its mission better. It is a reference tool for training and formative supervision. |
| 5 | Plan National de Développement Sanitaire 2011-2015 | Ministère de la Santé Publique - RDC (2011) | Health Policy document | It is a five-year plan for implementing the strategy for strengthening the health system. It includes, among other things, the situational analysis of the health sector reform and the action programme for five years. |
| 6 | Gouvernance et soutien provincial au district de santé en RD-Congo | Kahindo M. et al. (2011) | Journal article | This article describes good governance practices at the provincial health administration in North Kiivu that have contributed to strengthening support to health districts and improving their performance regarding coverage and access to health care and services. |
| 7 | Fascicule Métier 1 de la DPS : "Appui technique aux zones de santé" | Bonami M. et al. (2012) | Technical support report | This document results from participatory action research that led to restructuring the DRC's provincial health administration based on four roles: 1) support to health districts, 2) health information, communication and research, 3) inspection and control and 4) management of resources. It describes the definition and the overview of the first role of the provincial health administration: the support to health districts. |
| 8 | Recueil des normes de fonctionnement des structures sanitaires de la zone de santé en République Démocratique du Congo | Ministère de la Santé Publique - RDC (2012) | Health Policy document | This document is an update of the first edition (document #2). |
| 9 | Description des emplois au niveau provincial | Ministère de la Santé Publique - RDC (2013) | Health Policy document | This document is a compendium of job descriptions within the provincial health administration office, including that of the head of the technical support office for health districts. |
| 10 | Rôles exercés par le niveau intermédiaire du système sanitaire en République Démocratique du Congo : représentations des acteurs | Kahindo M. et al. (2014) | Journal article | This article reports on the stakeholders' representations of the role of the provincial health administration in the DRC. It provides insightful information on the subsidiary model of provincial health administration, which is well adapted to supporting health districts. |
| 11 | Réformes des structures intermédiaires de santé en République Démocratique du Congo | Kahindo M. et al. (2014) | Journal article | This article reports on the process and results of action research on restructuring the provincial health administration in the DRC. It provides relevant information on the optimal organisation and functioning of the provincial health administration, which guarantees effective technical support to the health districts. |
| 12 | Représentations d'acteurs sur l'assistante technique en République Démocratique du Congo | Ribesse N. et al. (2015) | Journal article | This article presents a study in the Democratic Republic of Congo aiming to identify perceptions about two types of technical support providers for health system strengthening: long-term technical assistants (agents of development agencies) and provincial health administration staff (agents of the Ministry of Health). |
| 13 | Accompagnement des équipes des zones de santé par le niveau intermédiaire : De la supervision au coaching intégrant le paradigme d’apprentissage | Kahindo M. (2016) | Technical support report | This document argues for a shift from supervision to coaching in supporting health districts by the provincial health administration. It outlines the limitations of the supervision approach. It describes the coaching process focusing on the coach's attributes, the learning process and the supportive interaction between the coach and the coachees. |
| 14 | Plan National de Développement Sanitaire 2016-2020 : vers la couverture sanitaire universelle | Ministère de la Santé Publique - RDC (2016) | Health Policy document | It is a five-year plan for implementing the strategy for strengthening the health system. It includes, among other things, the situational analysis of the health sector reform and the action programme for five years. |
| 15 | Normes, directives et instructions nationales de supervision intégrée en République Démocratique du Congo | Ministère de la Santé Publique - RDC (2016) | Health Policy document | This document outlines standards and guidelines of supportive supervision at all levels of DRC's health system: from national to provincial level, from provincial to district level, from district office to health facilities. |
| 16 | Plan National de Développement Sanitaire recadré pour la période 2019-2022 : Vers la couverture sanitaire universelle | Ministère de la Santé Publique - RDC (2018) | Health Policy document | It is a four-year plan for implementing the strategy for strengthening the health system. It includes, among other things, the situational analysis of the health sector reform and the action programme for the four years. |
| 17 | Soutien du niveau intermédiaire du système de santé aux équipes cadres des districts sanitaires : cas de la ville de Lubumbashi, République Démocratique du Congo | Chuy K.D. et al. (2020) | Journal article | This article report on the assessment of the coherence and relevance of the interactions between support from the provincial health administration and the maturity of district health management teams to lead their health districts in Lubumbashi. Stakeholders' perceptions of this provincial support are reported. |
| 18 | Soutien du niveau intermédiaire du système au district de santé : perceptions des équipes de district de santé du Nord Kivu à l'est de la RDC | Kahindo M. et al. (2021) | Journal article | This article describes the perceptions of health district management teams regarding to the support from the provincial health administration staff in the province of Nord-Kivu in eastern DRC. |
| 19 | Rapport des missions d’évaluation participative et d’accompagnement au changement des structures organisationnelles du niveau intermédiaire de la Province du Bas-Congo et de la Province de la Tshopo (Province Orientale) | Bonami M. et Godelet E. (2012) | Technical support report | This report outlines a technical support mission that aimed to strengthen the health system through a participatory reorganisation at the provincial level in the Bas-Congo and Tshopo to feed into the process of reflection on strengthening the health system in the DRC. |
| 20 | Constitution de la République Démocratique du Congo | Présidence de la République RDC (2006) | Policy document | This document is the constitution of the DRC which establishes decentralisation as a method of governance and distributes power between the national and provincial levels. |
| 21 | Formalisation des dispositifs mis en place à la division provinciale de la santé du Nord-Kivu dans le cadre de la SRSS | Bonami M. et Godelet E. (2009) | Technical support report | This technical document provides a basis for reflection on the restructuring of the Provincial Health Administrations in the DRC. It aims to describe the collective working methods gradually implemented throughout the 2000s within the North Kivu Provincial Management Team. |
