# Supplementary material for: The role of provincial health administration in supporting district health management teams in the Democratic Republic of Congo: eliciting an initial programme theory of a realist evaluation
Source: Health Res Policy Syst. 2024 Feb 20;22:29. doi: 10.1186/s12961-024-01115-9 (PMC10880215; doi:10.1186/s12961-024-01115-9)
Supplement: Supplementary file 2 — Additional file 2. Interview guide for programme designers. [file 12961_2024_1115_MOESM2_ESM.docx]

Additional file 2. Interview guide for program designers

| Elements | Actions /Questions/Remarks |
| --- | --- |
| Introduction | Arrival and installation.  Explain the purpose of the interview.  Explain the process of the interview (see information sheet)  Guarantee anonymity and confidentiality.  Ask for the interview to be recorded and notes to be taken.  Obtain the informed consent.  Install and check the recording material. |
| Interview beginning | Thank you for participating in this interview. To begin, I would like to ask you some general questions about your involvement in the reform process of the provincial health administration (PHA). |
| General questions | Q: Could you provide a brief overview of your responsibilities and role in restructuring the PHA in the Democratic Republic of Congo (DRC)  Q: Could you provide a brief overview of the process of this reform? Probing: motivation, agenda setting, main stages, actors involved, implementation process. |
| Transition | Let us talk now about the expected outcomes of the PHA reform. |
| Expected outcomes of the PHA reform | Q:  To effectively support the development of health districts in the DRC, how should a PHA organise and operate in accordance with the reform?  Q: What skills should the management team develop to manage a health district effectively in the DRC?  Probing: management, leadership and other skills.  Q: How should a district health management team (DHMT) organise itself and function so that it can fully play its role in steering the health district in the DRC? |
| Transition | Let us look at the process of technical support to the health districts. |
| Process of technical support to DHMT | Q: In your opinion, what would be the profile of an ideal provincial coach?  Probing: training, qualifications, skills and individual qualities (knowledge, know-how and interpersonal skills)?  Q:  Can you provide details on the content and procedure for a technical support visit by a provincial coach to the DHMT? This includes what should be done before, during, and after the visit.  Q: What do you think are the factors that can have a positive or negative effects on technical support for DHMTs at the individual, institutional, and environmental levels?  Q: As the provincial coaches hold a hierarchical position within the PHA office, what kind of relationship should they have with the DHMTs?  Probing: hierarchical, technical, other?  Q: Do these relationships have a positive or negative impact on the learning process? If they have a negative effect, what measures can be taken to address it?  Q: Aside from providing technical support, what other roles can a provincial coach assume in working with DHMTs? How do these roles help in enhancing the DHMTs' managerial capabilities? |
| Announcement of the end | We're almost at the end of our interview, but I have one or two final questions before we finish... |
| Miscellaneous | During this interview, you provided valuable information regarding the process and expected outcomes of technical support from provincial coaches to DHMTs.  Q: Is there anything else regarding technical support that you would like to share with me? If there is, we have enough time to discuss it right now.  Q: Do you have any questions you would like to ask me? |
| End of the interview | Thank you for being available. I would like to assure you that your answers are confidential. If I need more information, I will contact you without hesitation. |
